# A Compact Implantable Multiple-Input-Multiple-Output Antenna for Biotelemetry and Sensing Applications

**DOI:** 10.3390/s25113323

**Published:** 2025-05-25

**Authors:** Jamel Smida, Mohamed Karim Azizi, Anandh Sam Chandra Bose, Mohamed I. Waly

**Affiliations:** 1 College of Applied Science, AlMaarefa University, Riyadh 11597, Saudi Arabia; asam@um.edu.sa; 2 Microwave Electronics Research Laboratory, Faculty of Sciences of Tunis, University Tunis El Manar, Tunis 2092, Tunisia; medkarim.azizi@gmail.com; 3 Higher Institute of Multimedia Arts of Manouba, University of Manouba, Manouba 2010, Tunisia; 4 Department of Medical Equipment Technology, College of Applied Medical Sciences, Majmaah University, Al Majmaah 11952, Saudi Arabia

**Keywords:** antenna sensor, implantable antenna, gain, tumor

## Abstract

Gastrointestinal (GI) tract diseases are among the most common diseases in the world, resulting in more than 8 million deaths. The majority of these deaths occur due to cancer or tumors. Early detection of these tumors can greatly lower the mortality rate. In this work, an implantable multiple-input-multiple-output (MIMO) antenna sensor is constructed for GI tract devices to detect the tumor. The implantable MIMO antenna sensor has two embedded antennas, each operating at 915 MHz. Both elements of the system are placed 0.6 mm apart from each other (edge-to-edge). The volume consumed by this design is measured to be 7 × 7 × 0.25 = 12.25 mm^3^. It occupies a very small volume due to miniaturization achieved using meandered resonating structures and a high-permittivity substrate. It maintains stable radiation performance (gain = −26.2 dBi at resonance). The antenna units are decoupled by maintaining a proper gap between them and adding a slot on the bottom side. An isolation level greater than 28.7 dB is achieved using these approaches. Since the MIMO system utilizes two antenna elements, its effectiveness is verified using MIMO parameters. At SNR = 20 dB, the channel capacity reaches 8.75 bps/Hz. The proposed antenna ensures high channel capacity and enables seamless communication while simultaneously acting as a sensor to monitor internal changes in the observed region. The frequency response change with variations in the permittivity of human tissue, enabling its sensing capability. Moreover, the antenna sensor maintains stable radiation and S-parameter performance throughout the sensing mechanism. Thus, the proposed solution is suitable for biomedical implants requiring both high-data-rate communication and sensing.

## 1. Introduction

Recently, biomedical implants, biomedical sensors, and the Internet of Medical Things (IoMT) have become crucial for healthcare systems, as they play a vital role in diagnosing and regulating the inner parts of the human body [1,2,3]. Biomedical implantable devices have several important applications, including but not limited to leadless pacemakers [4], capsule endoscopy [5], intra-oral tongue drive systems [6], blood and glucose monitoring [7], intracranial pressure monitoring [8], and nerve stimulation [9]. Depending on the application, most of these devices are embedded with printed circuit boards (PCBs), sensors, batteries, cameras, and, most importantly, implantable antennas. Among them, the wireless capsule endoscope is a device that monitors the internal organs of the gastrointestinal (GI) tract by transmitting concurrent videos/images. These implantable devices are equipped with an optical dome, camera, PCB, sensors, batteries, and various electronic and radio-frequency (RF) components. Implants that travel through the GI tract remain there for an extended period to capture and transmit data over a wireless channel. It is crucial to ensure that the batteries have sufficient power. The battery life primarily depends on the microelectronic circuitry; if the circuitry consumes excessive power, the battery drains quickly. The integrated sensors in the circuitry draw significant power. This power consumption can be reduced by introducing an antenna that can perform both wireless communication and sensing.

Recently, several implantable antenna sensors have been introduced that function both as sensors and as wireless communication devices. In [10], the authors proposed a compact implantable antenna sensor to detect electrical changes in breast tissue. This implantable antenna sensor has a volume of 13.2 mm^3^ and operates at the 2.45 GHz band. Moreover, it exhibits reasonably good gain and radiation performance. The sensitivity performance is verified with the help of the frequency response of the antenna. With dielectric constant decreasing (indicating tumor growth), the reflection coefficient moves to higher bands. For every change of 10 in permittivity, a frequency shift of 9–18 MHz is observed. In [11], an antenna sensor is designed for integration into a GI tract implant. This antenna sensor occupies a highly compact volume of 2.97 mm^3^. It demonstrates a high gain and better radiation characteristics. The antenna sensor consists of multiple layers (a total of three) to accommodate the patches and ground plane. This sensor’s real-time performance was tested in a portion of minced meat taken from pork meat. Both of these sensors operate on the same principle. The area surrounding the antenna sensor acts as a capacitor. As permittivity decreases, capacitance decreases, causing the antenna’s resonance to shift toward higher frequencies. However, all the antenna sensors discussed above operate using a single port. As a result, they have limited channel capacity despite their good sensing attributes. They are also more susceptible to multipath propagation and lack spatial diversity. Modern wireless implant devices are equipped with high-resolution mini-cameras that capture high-definition images and videos. These advanced implantable devices transmit data to external nodes at data rates exceeding 78 Mbps. However, single-port antennas cannot support such high data rates [12,13]. To address this limitation, implantable MIMO antennas have been introduced [14,15,16,17,18,19]. MIMO systems significantly enhance the channel capacity of wireless communication without requiring an additional frequency spectrum. However, they consume more space due to there being more interconnected elements [20,21,22].

In [14], a high-speed telemetry-enabled antenna system is designed which has a high isolation level. The isolation is improved by introducing a neutralization line. In [15], the authors designed a scalp-implanted MIMO antenna with broadband behavior. Moreover, a via is used to isolate the four activated MIMO units. In [16], the authors used coplanar waveguide (CPW)-enabled resonators to construct a three-dimensional implantable antenna, enabling polarization and pattern diversity. The disconnected ground planes in the system contribute to a high isolation value. Similarly, a MIMO antenna system consisting of two units is designed in [17], operating at 403 MHz. In [18], the authors investigated coupling by varying the distance between the antennas. The authors in [19] designed a MIMO antenna with four elements operating at 2.45 GHz. They introduced electromagnetic band gaps (EBGs) to enhance isolation at 2.45 GHz. In [20], the authors used a combination of meandered resonators and a defected ground plane to design a wideband implantable MIMO antenna. In [21], a MIMO antenna consisting of two elements is designed to operate at 433 MHz, where compactness is achieved using capacitive slots. The authors in [22] have used two meandered resonators to construct a very compact MIMO antenna. In [23], the authors designed a very small implantable MIMO antenna for dual-frequency resonance. They employed several techniques to improve isolation, including a thin substrate and a discrete inductor. The combined effect of these techniques resulted in very low mutual coupling values (−35.85 dB at 915 MHz and −31.6 dB at 2.45 GHz). Similarly, the authors in [24] designed a three-dimensional MIMO antenna to enable radiation pattern diversity, making it well-suited for moving implants. This antenna is highly compact, occupying only 118.75 mm^3^ in volume, and operates at 915 MHz and 2.45 GHz. It maintains high isolation by positioning the antennas orthogonally to each other. In [25], the authors used meandered resonators to construct a MIMO design. The MIMO antenna system maintains good peak gain and efficiency. All antennas discussed in this paragraph lack sensing ability. In the literature, implantable antenna sensors are either designed as single-port systems or as implantable MIMO antenna systems, which lack sensing functionality.

To address the limitations of antenna sensors (single-port with low channel capacity) and implantable MIMO antennas (no sensing functionality), this work presents a dual-channel implantable MIMO antenna sensor. The proposed approach enables high-speed wireless communication with high channel capacity while also functioning as a sensor to detect changes in human tissue. The suggested system features two embedded implantable antennas, each operating at the 915 MHz frequency band.

## 2. Implantable MIMO Antenna Sensor Design

**Figure 1:**
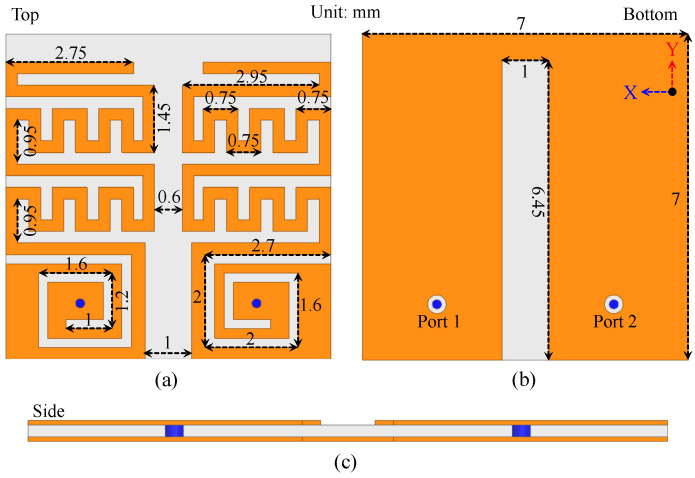
Structure of the implantable MIMO antenna sensor.

This multi-antenna system has a couple of embedded antenna units, each made of spiral and meandered resonators (Figure 1). The final optimized design and its complete dimensions are also given. The proposed design is simulated and fabricated on a high-permittivity Rogers 3010 substrate, known for its low tangent losses and high dielectric constant, which helps reduce the antenna volume. The spiral and meandered resonators are placed on top, and ground metal is positioned below the laminate. Two antenna units are placed face-to-face with a 0.6 mm edge-to-edge gap. The overall volume occupied by the antenna sensor is 7 × 7 × 0.25 = 12.25 mm^3^. This compact volume is achieved through miniaturization techniques using meandered resonating structures and a high-permittivity substrate. Both antenna units are excited using a coaxial probe, where miniaturization is further enhanced by the meandered and spiral geometry. Additionally, the high dielectric constant of the substrate contributes to miniaturization. Both antenna units operate at the same frequency band.

### 2.1. Simulation Site and Position of Implant

The surrounding environment of an antenna greatly impacts its S-parameters and radiation performance. Each antenna has a different installation site and location. Thus, it is always good practice to design and optimize an antenna in the environment where it will operate. Moreover, it is important to add dummy devices or real ones near the antenna to analyze their impact.

**Figure 2:**
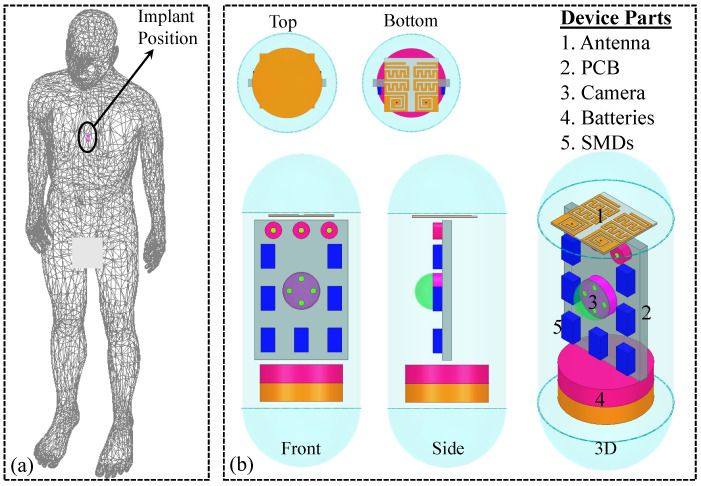
(**a**) Location of the implant inside the human body (Stomach region and at depth of 55 mm). (**b**) Device architecture of implant where implantable MIMO antenna sensor operates.

This antenna is designed for a wireless capsule device intended for implantation in the human stomach. The primary function of such implants is to transmit recorded images and videos to external hardware. Considering these factors, the antenna sensor is optimized within a human stomach model with implant depth of 55 mm. Initially, the full human phantom from High-Frequency Structure Simulator (HFSS) is imported. Then, frequency-dependent electrical properties are assigned to it. The electrical properties are selected based on [26]. The electrical properties of stomach tissues at different frequencies are given in Table 1. The antenna is subsequently added in the stomach and optimized for optimal performance and the location is shown in Figure 2a. Specifically, the antenna is optimized for minimal reflection coefficient (S_11_ < 10 dB) at 915 MHz, and stable impedance matching within the stomach environment. The optimization parameters included the copper trace widths and lengths, the gap between the resonators, and the dimensions of the grounded slot. These are iteratively adjusted using the parametric sweep and optimization tools available in the simulation software (HFSS). A terminal mode is selected from the solver modes. The frequency range is selected from 0.6 GHz to 2 GHz with a total of 401 points. The frequency sweep solver of interpolating is chosen. The mesh size is kept as low to have more realistic results.

### 2.2. Implantable Device Construction and Its Parts

Antenna geometry, PCB, device components, and casing significantly contribute to antenna performance. Their placement and location can either degrade or improve the antenna’s performance. For example, an extended copper layer beneath the antenna geometry can increase the gain. Therefore, optimizing radiating or sensing antennas within the surroundings of these components is crucial.

These implantable devices are equipped with an optical dome, camera, PCB, batteries, and various electronic and RF surface-mounted devices (SMDs). Thus, this system is also executed in the presence of these elements. Final design of the implantable device and its circuitry is shown in Figure 2b. The electronic and RF parts are located in a capsule made of polylactic acid (PLA) [dielectric constant = 2.8, loss tangent = 0.006]. This specific material is selected because it is biocompatible, a crucial attribute for biomedical implants. The suggested implant, which integrates the antenna, is 25 mm long and has a diameter of 12.8 mm. The capsule casing material has thickness of 0.25 mm. The PCB is 10 mm wide, 15 mm long and 1 mm thick. Each battery has a radius of 4.5 mm and height of 2 mm.

### 2.3. Design Evolution of a Single Unit of the MIMO Antenna Sensor

**Figure 3:**
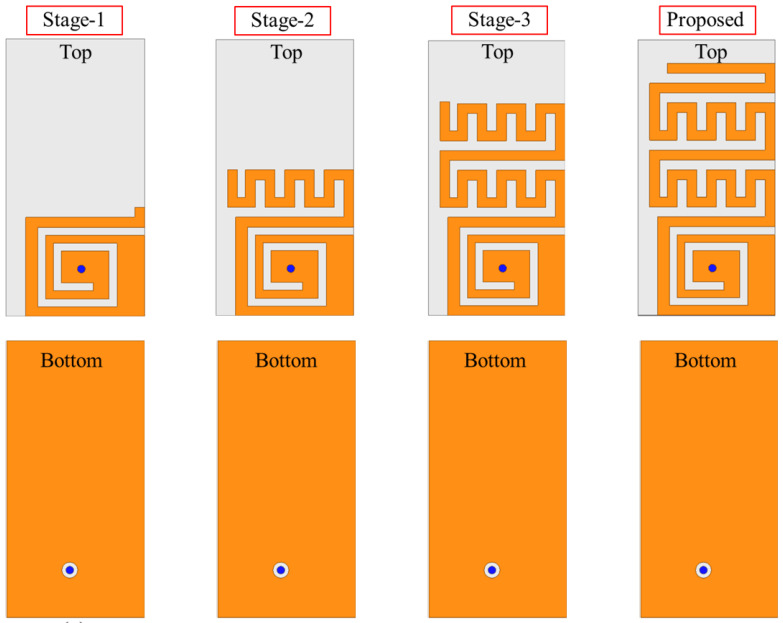
Design stages of implantable antenna sensor.

**Figure 4:**
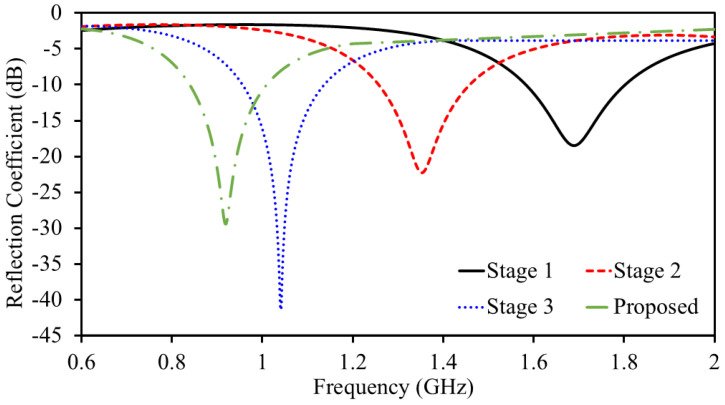
Design evolution stages’ reflection coefficient.

Before finalizing the antenna for fabrication and measurement, it is first simulated and optimized for better performance. Initially, a single unit of the antenna is optimized and finalized. Several steps are involved in achieving better performance. After carefully optimizing a single element, two units of the MIMO antenna are realized to develop a MIMO system. The optimized parameters include the gap between the antennas and the dimensions of the slot on the ground plane. The optimization goals are to achieve a reflection coefficient below −10 dB within the desired bandwidth, improved impedance matching, and an isolation level greater than 25 dB. A step-by-step analysis of the single antenna element is portrayed in Figure 3 and Figure 4.

Initially, a portion of the Rogers 3010 substrate is designed followed by a ground at lower portion. A spiral radiator is then designed. A coaxial feed with a 50 Ω impedance is used to excite the spiral radiator, with the ground plane serving as the reference. A spiral geometry is chosen to ensure a long radiator within a small space, achieving lower resonance in a confined area to create a miniaturized structure. The initial parameters are calculated using the following relationship:

(1) $$L_{res}=\frac{c}{4\times f\sqrt{\epsilon_{r}}}$$

where $L_{res}$ shows the dimensions of the spiral radiator, and *f* represents the operating frequency.

In Stage-I, the antenna resonance is noted to be at 1692 MHz. The reflection coefficient value at the resonance is found to be −18.39 dB. In Stage II, a meandered resonator is attached to the end of a spiral resonator, increasing the overall resonator length. As the length increases, the frequency shifts to a lower value, as verified by Equation (1). The adjacent arms of the meandered resonator introduce additional capacitance, which contributes to antenna miniaturization by lowering the resonant frequency. The role of this additional capacitance in altering the resonance can be verified with

(2) $$v_{p}=\frac{1}{\sqrt{L_{res}C_{res}}}=\frac{v_{c}}{\sqrt{\epsilon_{eff}}}=f\lambda_{g}$$

where $v_{p}$ is the slow-wave propagation velocity, $L_{res}$ and $C_{res}$ are reactance of the radiating structure, *f* is the operational resonance. It is evident from the above equation that miniaturization occurs when the reactance of the radiating structure increases. In our scenario, the added capacitance enhances the total reactance, which contributes to an improved miniaturization ratio. The adjacent arms of the meandered resonator introduce additional capacitance, which contributes to antenna miniaturization by lowering the resonant frequency because it further enhances the overall reactance.

**Figure 5:**
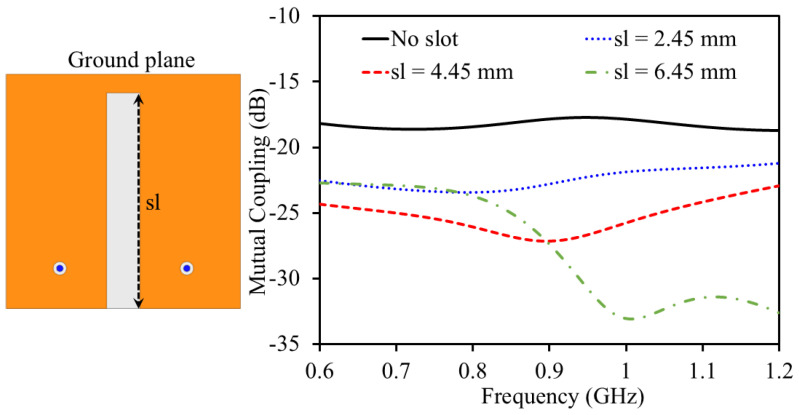
Impact of the slotted ground plane on the mutual coupling.

In Stage-II, the antenna resonance is noted to be at 1356 MHz. The reflection coefficient value at the resonance is found to be −22.23 dB. In Stage-III, a further meandered resonating structure is added to the previous one to extend the resonator length. This not only increases the resonator length but also adds additional capacitance. As mentioned earlier, the length of the resonator has an inverse relationship with frequency; therefore, the resonance shifts to lower frequencies. Moreover, the meandered geometry adds additional capacitance, which further lowers the frequencies according to the slow-wave equation. In Stage-III, the antenna resonance is noted to be at 1042 MHz. The reflection coefficient value at the resonance is found to be −41.34 dB. In Stage-IV (Proposed), the resonator size is further elongated to resonate at 915 MHz. In Stage-IV (Proposed), resonance is noted to be at 0.915 GHz and it has a bandwidth of 188 MHz (830–1018 MHz). The reflection coefficient value at the resonance is found to be −29.39 dB. Once one unit element of the MIMO system is finalized, a second one is placed at a distance of 0.6 mm (edge-to-edge) to construct a two-element implantable MIMO antenna sensor. Both unit elements share a same ground plane and a substrate which is desirable for a system level implementation. To improve the isolation between the two antenna elements, a small slot was introduced in the ground plane separating them. To evaluate the impact of the ground plane on mutual coupling, several simulations were conducted. Initially, a full ground plane (i.e., no slot) was considered, followed by a parametric analysis of the ground slot, as shown in Figure 5. Slot lengths of 2.45 mm, 4.45 mm, and 6.45 mm were investigated. When no slot was present (full ground plane), the antenna exhibited a mutual coupling value of −17.78 dB at 915 MHz. Introducing a slot with a length of 2.45 mm reduced the mutual coupling to −22.69 dB at 915 MHz. Increasing the slot length to 4.45 mm further improved isolation, yielding a mutual coupling of −27.13 dB. Finally, with the proposed slot length of 6.45 mm, the mutual coupling was reduced to −28.7 dB at 915 MHz. This design choice plays a key role, especially in compact implantable systems wherein space is limited and the antennas are positioned close together. Without any isolation technique, electrical currents flowing across the ground plane can easily pass from one antenna to the other. This interaction, known as mutual coupling, negatively affects performance by reducing isolation, increasing signal interference between the channels, and distorting the radiation patterns. The ground plane slot acts as a barrier that limits this current flow, essentially creating a high-resistance path between the antennas. By interrupting the surface current, the slot helps suppress unwanted interactions and significantly improves the isolation. Importantly, this approach does not depend on extra decoupling circuits or external components, making it especially suitable for miniaturized biomedical implants where simplicity, space, and compatibility with human tissue are all crucial.

### 2.4. Impact of Capsule Device on S-Parameters and Directivity

**Figure 6:**
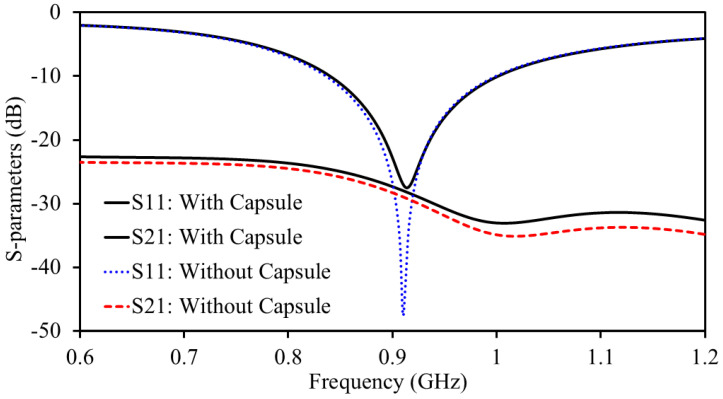
S-parameters of the antenna with and without the capsule device.

**Figure 7:**
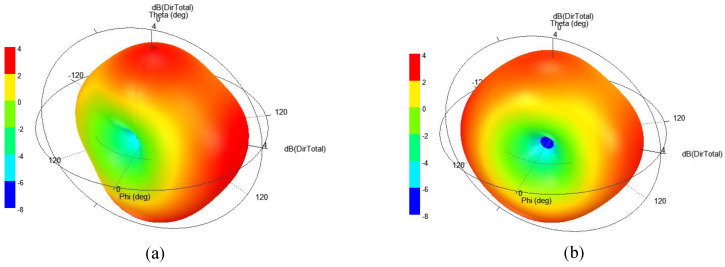
Directivity plots of the antenna (**a**) with capsule device, and (**b**) without capsule device.

In the proposed MIMO antenna design, a PCB is placed vertically beneath the antenna structure. Extensive full-wave simulations were carried out to assess the impact of the PCB on the antenna’s performance metrics, including impedance matching, mutual coupling, gain, and radiation pattern. These simulations demonstrated that the presence of the PCB has a minimal to negligible effect on the antenna’s performance. The proposed antenna has a complete ground plane, which restricts coupling between the antenna and capsule components. Therefore, the antennas’ attributes were less impacted. The S-parameters of the antenna with and without the capsule device are shown in Figure 6. It can be observed that only a minimal impact has been observed from the capsule device. Moreover, 3D directivity pattern of the antenna (Antenna-II) with and without being integrated in the capsule device are shown in Figure 7.

## 3. Results and Discussions

**Figure 8:**
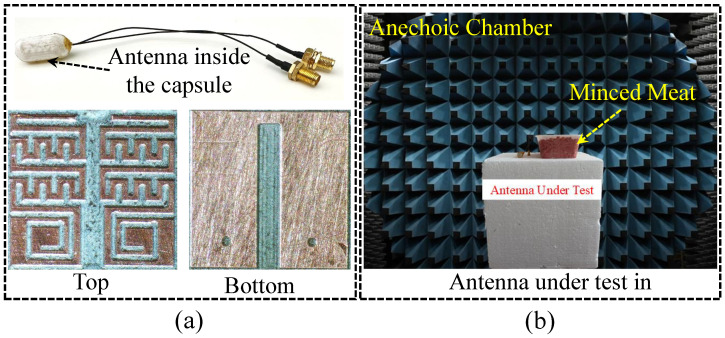
(**a**) Photo of the fabricated antenna. (**b**) Antenna measurement in anechoic chamber.

The effectiveness was investigated through simulations followed by the practical measurements. Therefore, the single-unit of the MIMO antenna sensor is designed at the beginning. Once the targeted goals are achieved for the design of one structure, the second unit is placed at a distance of 0.6 mm (edge-to-edge) to construct a two-element implantable MIMO antenna sensor. Then, a cut in the ground plane is added to block traveling of leaked current to the second one to enhance isolation level. After a fully workable implantable MIMO antenna sensor design is executed in phantom, a workable prototype of the same is constructed. For the prototype, initially a Gerber file for the top, bottom and board outline is prepared for the laser machine. First, copper layers are designed over Rogers 3010 substrate. Once the top and bottom layers are fabricated, a high laser beam is used to cut the boundaries of the prototype. The coaxial cables for antenna sensor testing are soldered in the ports region. The prototype is shown in Figure 8. The proposed antenna sensor is simulated inside a dummy capsule device. To obtain a more realistic performance comparison, the antenna sensor is tested inside the device as well. To achieve this, a 3D capsule device is made using a 3D printer. Then, a mini-PCB and batteries are placed alongside the fabricated antenna sensor. To measure the antenna sensor, it is embedded within the meat. The electrical properties of the meat are tested using a vector network analyzer to assess its resemblance to the simulation environment. Once confirmed, measurements are performed. Before measuring the antenna sensor, all equipment is calibrated for best results.

**Figure 9:**
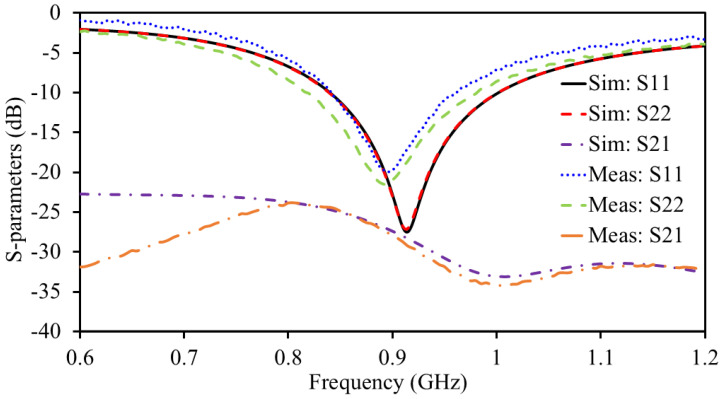
Simulated and measured S-parameters.

The S-parameters are measured through the well-calibrated vector network analyzer, where both reflection coefficients (S_11_ and S_22_) and isolation level (S_21_) are measured, as presented in Figure 9. The simulated antenna resonance (S_11_ and S_22_) is noted to be at 0.915 GHz and it has a bandwidth of 188 MHz (830–1018 MHz), as presented in Figure 9. The simulated value for both S_11_ and S_22_ at the resonance is found to be −29.39 dB. A simulated isolation level greater than 28.7 dB is found within the resonance. The resonance of the measured S_11_ and S_22_ is found to be at 904 MHz. The measured bandwidth is found to be 134 MHz (S_11_) and 165 MHz (S_22_). The measured reflection coefficient value for S_11_ and S_22_ at the resonance is found to be −19.95 dB and −21.52, respectively. A measured isolation of 27.8 dB is found on the resonance frequency.

**Figure 10:**
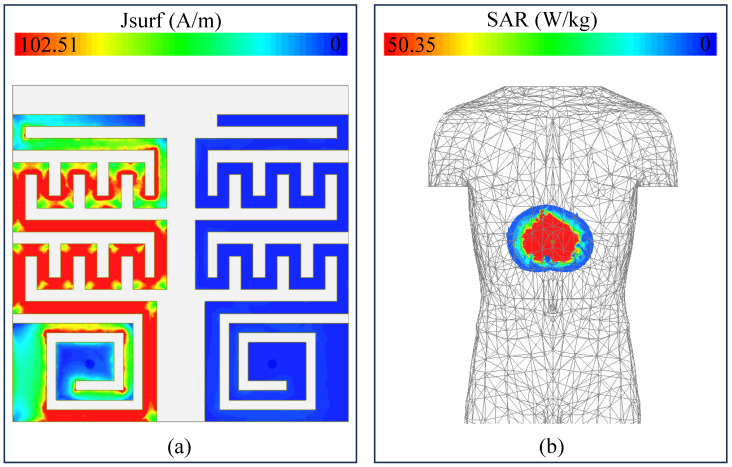
Simulated (**a**) surface current density at 915 MHz and (**b**) SAR at 915 MHz.

The current density of the MIMO sensor is illustrated in Figure 10a. In this study, one unit is activated with a 1W power while the second unit is passive. It can be seen that the current is majorly positioned over the active unit while the passive unit receives almost negligible amount of leaked current. This shows that both elements are highly isolated as a minimal amount of surface current is being received by the passive unit. This isolation is mainly attributed because of the proper distance between the resonators, ultra-thin substrate (thickness = 0.25 mm), and slot in the ground plane.

Apart from optimal performance in terms of S-parameters and radiation characteristics, ensuring safe electromagnetic exposure inside the human body is also essential. Implantable devices use antennas to transfer data from inside the body to external nodes through electromagnetic waves radiated by the antenna structures. These waves interact with human body tissues, which can be damaged if the exposure level is too high. The IEEE introduced the specific absorption rate (SAR) to define the exposure received from wireless waves. These safety standards suggest that the exposure (SAR) must not exceed some standard levels. For example, the most used standard is 2 W/kg to ensure safe operation [27]. To evaluate the SAR level, both ports are activated. Under these conditions, an SAR value of 50.35 W/kg is observed at 915 MHz, as shown in Figure 10b. The SAR value vary with the input power and has a direct relationship with it. This SAR value is evaluated based on 1 W of input power. In practical scenarios, the maximum power is restricted to −16 dBm. Based on −16 dBm input power, the SAR value is 0.00126 W/kg, which is well below the standards. Given this constraint, the device remains suitable for use.

**Figure 11:**
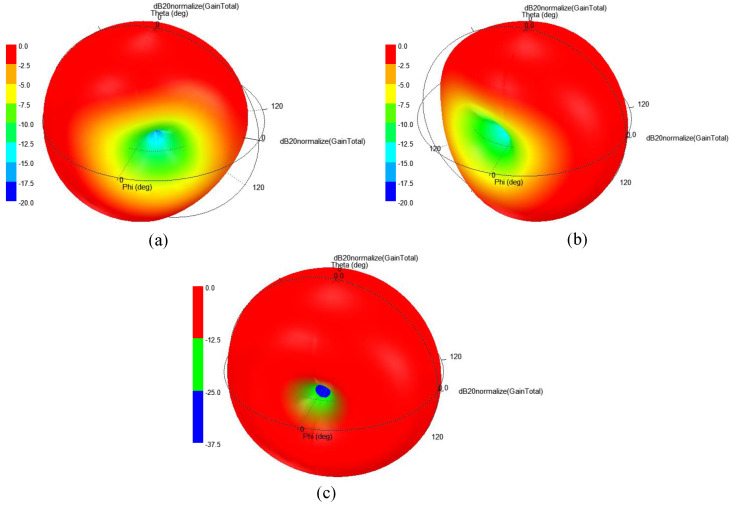
Normalized 3D radiation patterns of (**a**) Antenna-I, (**b**) Antenna-II, (**c**) combined pattern of both antennas.

The 3D far-patterns of the antenna (Antenna-I, Antenna-II, and combined pattern of both antennas) are evaluated, as shown in Figure 11. The radiation patterns are almost omni-directional, which are very useful for wireless capsule implant devices. Devices having such beams are useful as they travel along the GI tract and randomly change their directions. In case of such patterns, the data can be delivered at any direction without any disruption. The simulated gain at 915 MHz is found to be −26.2 dBi. For the same frequency, the measured gain is noted as −27.7 dBi.

### 3.1. Link Margin Analysis

The wireless capsule implant travels in the GI tract and collects information through installed sensors and cameras. This important information is then sent to external nodes through integrated implantable antennas. It is always necessary to see the ability of the capsule implant to deliver data efficiently. This ability can be determined by accessing the transmitting range of the device. Link margin is one of the best ones to determine the range. In our design, the proposed antenna acts as a sensor as well as a transmitting antenna. Thus, the link margin analysis of the proposed implantable MIMO antenna sensor is performed to investigate its performance in terms of transmitting range. We considered this structure as a transmitter because it is integrated within the capsule implant. Moreover, we considered a dipole antenna with a gain of 2.1 dBi as a receiving node. The link margin is calculated as

(3) $$LM=P_{av}-P_{rc}$$

where

(4) $$P_{av}=P_{t}+G_{t}+G_{r}-10log_{10}{\left(\frac{4\times \pi d}{\lambda }\right)}^{2}-N_{\circ }$$

and

(5) $$P_{rc}=E_{b}/N_{\circ }+10log_{10}\left(B_{r}\right)+G_{d}$$

**Figure 12:**
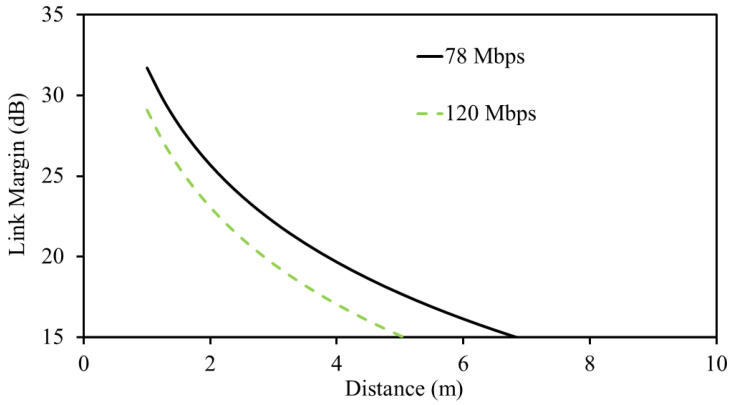
Calculated wireless link margin at 915 MHz.

In the above equations, LM is the link margin, $P_{av}$ is the available power to extract data from, $P_{rc}$ is the actual received power by the dipole antenna placed in external node, $P_{t}$ is the transmitted power from the capsule implant which is selected based on the IEEE standard, $G_{t}$ is th gain of the transmitter antenna (in our case, the transmitter antenna is the MIMO antenna sensor), $E_{b}/N_{\circ }$ is the phase-shift-keying whose value is set to be 9.6 dB, $G_{d}$ is fixing deterioration whose value is set to be 2.5 dB, and $B_{r}$ is the bit-rate. In this study, we fixed the bit-rate at 78 Mbps and 120 Mbps. These values are specifically selected to satisfy the requirement of next-generation capsule implants, where high-resolution cameras are installed [28]. For the link margin, the distance from the antenna sensor to the dipole antenna (installed in an external node) is varied and the value is recorded, as shown in Figure 12. Normally, a 0 dB link margin is enough for wireless communication. However, considering practical constraints and unexpected losses, we considered a link margin of 15 dB for seamless communication. With this specific value, the MIMO implantable antenna sensor can effectively communicate up to 6.77 m and 4.81 m at 78 Mbps and 120 Mbps, respectively.

### 3.2. MIMO Channel Parameters

**Figure 13:**
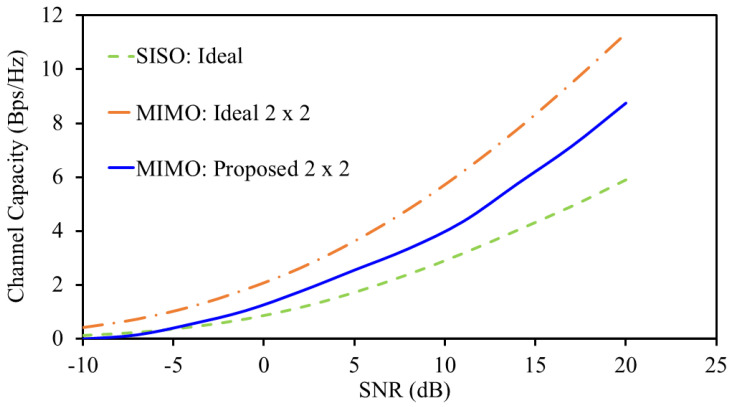
Channel capacity at 915 MHz.

The main difference between the conventional single-port antenna and multi-port MIMO antenna is the channel capacity. The conventional single-port antennas have less channel capacity compared to multi-element MIMO antennas. This due to the fact that they are less immune to multi-path propagation. In contrast, MIMO antennas are suitable for their ability to adjust to multi-path propagation. Thus, it is important to analyze the antenna performance in terms of channel capacity. Ideally, elements of the system should have zero correlation; however, this less possible in practical scenarios. Practically, there should be some sort of coupling between the elements. In addition, ideally, the number of elements in the system determine the channel capacity. Having a large number of elements in the system ensures high channel capacity. However, this estimation is based on the assumption that all elements are uncorrelated, which is very unlikely in practical life. In real-world applications, the channel capacity is determined by the number of elements along with the correlation between the elements. In fact, having a large number of elements with less correlation provides high channel capacity. The channel capacity for the proposed system is calculated using

(6) $$C=\log_{2}\left(\det\left[I+\left(\frac{SNR}{N}\right)HH^{*}\right]\right)$$

where *C* shows the channel capacity, *N* is the number of elements in the system, *I* is the identity matrix, *H* is the channel matrix, and H* is the conjugate of the channel matrix. The channel matrix contains important information on the elements of the system. They are normally calculated from the individual radiation characteristic of all elements as follows:

(7) $$H=\left[\begin{array}{cc} h_{11} & h_{12}\\ h_{21} & h_{22} \end{array}\right]=\left[\begin{array}{c} h_{ij} \end{array}\right]\;\mathrm{where}\;i,j\in \left\{1,2\right\}$$

where each channel coefficient $h_{ij}$ contains both gain (magnitude) and phase information. In this work, the channel capacities for the ideal single-port antenna, ideal dual-element MIMO antenna system, and proposed dual-element MIMO antenna system is calculated using (6), as shown in Figure 13. In an ideal dual-element MIMO antenna system, the channel capacity is based on the uncorrelated elements. However, in the proposed one, the correlation between the elements is taken into consideration. Based on (6), the ideal dual-element MIMO antenna system has better channel capacity than the other two. In addition, the proposed system has better channel capacity than the single-port antenna but lower than the ideal one. This is due to the fact that zero correlation between the elements is considered in ideal scenarios. At a signal-to-noise ratio of 20 dB, the channel capacity of the proposed system reaches 8.75 bps/Hz.

Another important parameter for MIMO channel characterization is the envelope correlation coefficient (ECC) of the system. This parameter shows the independence of each element of the antenna from the others in the system. It can be calculated using two methods. The first one uses S-parameters while the second one uses far-field patterns. In our work, we used the second method due to its more realistic values. The first method is more common for antennas having isotropic characteristics, which can only be identified in ideal scenarios. The following equation is used for this design:

(8) $$\mathrm{ECC}=\frac{{\left|\int \int_{4\pi }\left(A_{n_{i}}\left(\theta ,\phi \right)\right)\cdot \left(A_{n_{j}}\left(\theta ,\phi \right)\right)d\Omega \right|}^{2}}{\int \int_{4\pi }|A_{n_{i}}{\left(\theta ,\phi \right)|}^{2}d\Omega \cdot \int \int_{4\pi }{\left|A_{n_{j}}\left(\theta ,\phi \right)\right|}^{2}d\Omega }$$

where $\left(A_{n_{i}}\left(\theta ,\phi \right)\right)$ and $\left(A_{n_{j}}\left(\theta ,\phi \right)\right)$ are patterns for element 1 and 2 with Ω as solid angle. Based on this equation, an ECC value of 0.13 is found between element 1 and 2 at 915 MHz. The diversity gain is another vital parameter which depends on the ECC value. It can be computed as $10\sqrt{1-{ECC}^{2}}$, which shows a value of 9.99 dB.

### 3.3. Sensing Mechanism

The purpose of constructing this antenna sensor is to enable tissue sensing and wireless communication simultaneously. The wireless communication ability is verified in the previous parts of this paper, where various vital characteristics are verified. The sensing part of the proposed design is discussed in this section. In this section, we provided a theoretical explanation on how sensing occurs and why it occurs. Also, we performed simulations to verify the theory we already developed.

**Figure 14:**
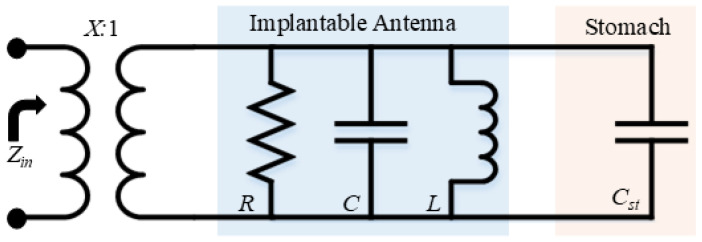
Lumped model of implantable antenna and the stomach of a human body.

When an implantable antenna is placed in the vicinity of tissue, the surrounding tissue behaves like a dielectric layer. This interaction creates a capacitance with the antenna. For ease of understanding, the overall antenna structure and surrounding tissues are modeled using lumped circuits. The equivalent circuit is shown in Figure 14, where RLC section shows the antenna behavior while the stomach tissue is represented with a capacitor ($C_{st}$). The factor *X* shows the impedance matching between the source and antenna. In this case, the source is a coaxial cable which has a 50 Ω impedance.

The capacitor ($C_{st}$) is the capacitance generated by the stomach tissue which depends on the permittivity of free space and stomach tissue, as shown by the following relationship:

(9) $$C_{st}=\epsilon_{\circ }\epsilon_{st}\frac{K^{\prime }\left(k_{1}\right)}{K(k_{1})}$$

where $C_{st}$ is the additional generated capacitance due to stomach loading, $\epsilon_{\circ }$ is the permittivity of free space, $\epsilon_{st}$ is the permittivity of stomach, and *K* is elliptical integral. As $\epsilon_{st}$ increases, the generated capacitance increases.

The equivalent circuit of a unit antenna sensor along with the capacitance generated by the stomach is shown in Figure 14. All elements of the circuit are highlighted for ease of understanding. Those resonators, whose resonance is below its fundamental frequency, can be represented with an inductor only [29,30,31]. With such simplification, the input impedance can be determined using

(10) $$Z=\frac{j\omega L_{equ}}{1-\omega^{2}C_{st}L_{equ}}$$

and

(11) $$\omega =\frac{1}{\sqrt{C_{st}L_{equ}}}$$

The input impedance *Z* of the antenna is influenced by its equivalent inductance $L_{equ}$, the angular frequency ω, and the induced capacitance $C_{st}$ resulting from the surrounding muscle tissue. As shown in (11), the antenna’s resonant frequency is determined by both $L_{equ}$ and $C_{st}$. Additionally, (9) highlights that $C_{st}$ is affected by the permittivity of the muscle tissue. Therefore, an increase in the tissue’s permittivity leads to a higher $C_{st}$, causing the resonant frequency to shift to a lower range.

The electrical characteristics of stomach tissue, particularly its permittivity, tend to change as gastric lesions develop and tumors grow [10,11]. As previously mentioned, any variation in tissue permittivity leads to a shift in the antenna’s resonant frequency. This frequency shift can be used as an indicator to detect the presence of gastric lesions or tumor growth.

**Figure 15:**
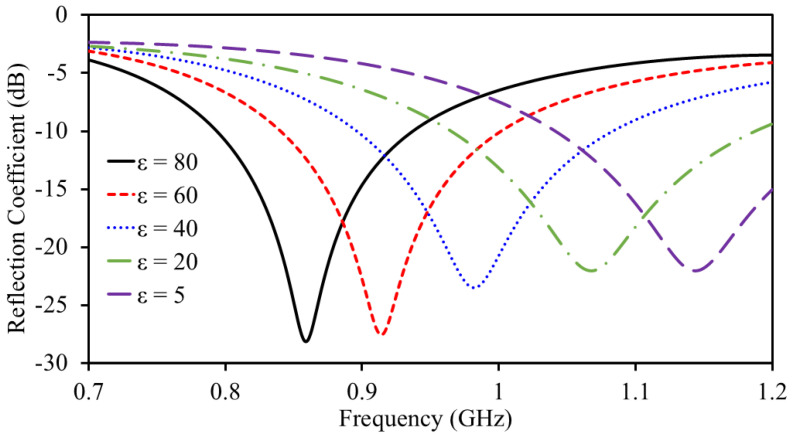
Sensing ability of the antenna sensor.

Figure 15 shows the antenna’s reflection coefficient (S_11_) as the stomach tissue’s permittivity changes. A decrease in permittivity causes the resonant frequency to shift toward higher values. For instance, the antenna resonates at 1.146 GHz (permittivity = 5). However, as the permittivity increases to 80, the resonant frequency drops to 0.86 GHz.

**Figure 16:**
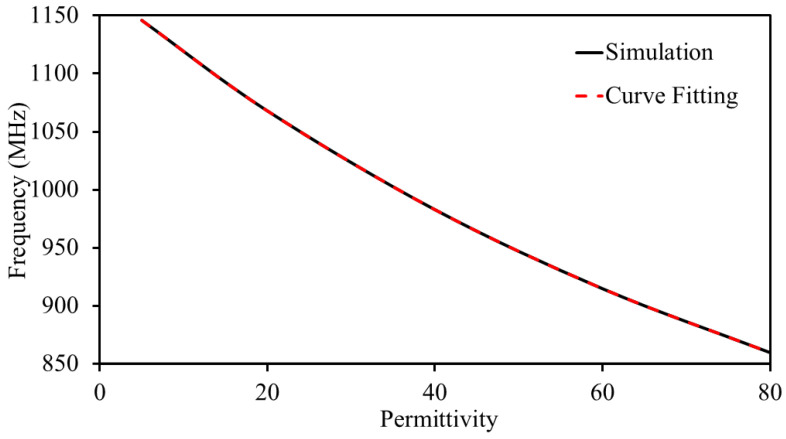
Simulated and curve fitting data of the antenna sensor.

The antenna demonstrates excellent matching across all conditions, with S_11_ consistently remaining matched. Figure 16 presents the obtained with simulations and curve fitting. The curve fitting is obtained by considering the S_11_. It is worth mentioning that both S_11_ and S_22_ are the same because both are similar in shape and size. The following polynomial regression equation shows the relationship of resonance with permittivity: $f=0.0209\epsilon_{r}^{2}-5.5653\epsilon_{r}+1172.4220$, achieving a high statistical accuracy with an $R^{2}$ value of 0.999. In this equation, *f* represents the antenna’s resonant frequency, while $\epsilon_{r}$ corresponds to the permittivity.

Given that the primary function of this antenna sensor is to communicate images/videos from within the body, it is essential to assess the antenna’s key performance metrics under various sensing conditions. The gain values are found to be −22.3 dBi when the permittivity is 5 and −29.1 dBi at permittivity of 80. The antenna is well-isolated for all permittivity ranges; it has isolation of better than 28.7 dB in all conditions. The MIMO channel parameters are also investigated at different sensing points (permittivity). The ECC of the antenna sensor is found to be less than 0.15 in all sensing points. Also, the DG is found to be more than 9.9 dB in all cases. Thus, it is proved that the proposed system is suitable for biomedical devices where sensing and data transfer is required at the same time but without an additional sensor.

The developed implantable MIMO antenna sensor system introduces a novel approach by integrating both high data capacity and sensing functions (see Table 2). Unlike conventional single-port antenna sensors with limited transmission capability, this design employs a MIMO setup to boost data rates and reliability. It also addresses the gap in existing implantable MIMO antennas, which lack sensing functionality, by incorporating sensing directly into the antenna. This combination enhances communication performance while enabling simultaneous real-time monitoring, offering a more efficient and multifunctional solution for biomedical use.

## 4. Conclusions

In this work, an implantable MIMO antenna sensor has been designed for GI tract devices to detect the tumor. The implantable MIMO antenna sensor has two embedded antennas, each operating at 915 MHz. Both elements of the antenna sensor were placed 0.6 mm apart from each other (edge-to-edge). The total volume consumed by the antenna sensor was found to be 12.25 mm^3^. It occupies a very small volume due to miniaturization that has been achieved using meandered resonating structures and a high-permittivity substrate. It has a stable radiation performance with a gain of −26.2 dBi at 915 MHz. The antenna units were decoupled by maintaining a proper gap between them and adding a rectangular slot on the ground plane. An isolation level greater than 28.7 dB was achieved using these approaches. The antenna sensor performance effectiveness was verified using the envelope correlation coefficient, diversity gain, and channel capacity. At an SNR of 20 dB, the channel capacity of 8.75 bps/Hz was found. The proposed antenna ensured high channel capacity and enabled seamless communication while simultaneously acting as a sensor to monitor internal changes in the observed region. The resonant frequency of the sensor shifted with variations in the permittivity of human tissue, enabling its sensing capability. Moreover, the antenna sensor maintained stable radiation and S-parameter performance throughout the sensing mechanism. Thus, the proposed solution was found to be suitable for biomedical implants requiring both high-data-rate communication and sensing.

## Figures and Tables

**Table 1:** Electrical properties of stomach tissue at different frequencies.

Frequency (MHz)	800	850	900	915	950	1000
Conductivity [S/m]	1.1447	1.1653	1.1867	1.1932	1.2088	1.2316
Relative permittivity	65.362	65.206	65.062	65.02	64.926	64.797
Loss tangent	0.3935	0.37793	0.36429	0.36053	0.35229	0.34167

**Table 2:** Comparison with state-of-the-art implantable antennas.

Ref.	Size (mm^3^)	No of Elements	Antenna Profile	Frequ. (MHz)	Isolation (dB)	Gain (dBi)	MIMO?	Sensing Ability?
[10]	13.2	1	Planar	2450	---	−8.4	No	Yes
[11]	2.97	1	Planar	2450	---	−9.7	No	Yes
[13]	9.01	2	Planar	915	29.7	−24.6	Yes	No
[14]	63.5	2	Planar	2450	22.93	−32.15	Yes	No
[15]	23.6	4	Planar	433	32.6	−28.3	Yes	No
[16]	3375	4	Cubic	2400, 5800	32	−18.5	Yes	No
[17]	307	2	Planar	402	25.6	−26	Yes	No
[18]	434.6	4	Planar	2400	15.9	−15.18	Yes	No
This work	12.25	2	Planar	915	28.7	−26.2	Yes	Yes

## Data Availability

All the data are available in the study.
